# Impaired autophagy in myeloid cells aggravates psoriasis-like skin inflammation through the IL-1β/CXCL2/neutrophil axis

**DOI:** 10.1186/s13578-024-01238-0

**Published:** 2024-05-04

**Authors:** Jinju Lee, Mi-Yeon Kim, Hyo Jeong Kim, Woo Sun Choi, Hun Sik Kim

**Affiliations:** 1 Department of Microbiology, Asan Medical Center, University of Ulsan College of Medicine, 88 Olympic-ro 43-gil, Songpa-gu, Seoul, 05505 Republic of Korea; 2 Stem Cell Immunomodulation Research Center (SCIRC), Asan Medical Center, University of Ulsan College of Medicine, Seoul, Korea

**Keywords:** Psoriasis, Autophagy, Myeloid cells, IL-1β, IL-17, Neutrophilic inflammation

## Abstract

**Background:**

Psoriasis is an inflammatory skin disease characterized by the hyperproliferative epidermal keratinocytes and significant immune cells infiltration, leading to cytokines production such as IL-1β, TNF-α, IL-23, and IL-17. Recent study highlights the critical role of IL-1β in the induction and activation of pathogenic Th17 and IL-17-producing γδ T cells, contributing to psoriasis. However, the mechanism underlying IL-1β dysregulation in psoriasis pathogenesis is unclear. Autophagy regulates IL-1β production and has a pleiotropic effect on inflammatory disorders. Previous studies showed controversial role of autophagy in psoriasis pathogenesis, either pro-inflammatory in autophagy-deficient keratinocyte or anti-inflammatory in pharmacologically autophagy-promoting macrophages. Thus, the direct role of autophagy and its therapeutic potential in psoriasis remains unclear.

**Methods:**

We used myeloid cell-specific autophagy-related gene 7 (Atg7)-deficient mice and determined the effect of autophagy deficiency in myeloid cells on neutrophilia and disease pathogenesis in an imiquimod-induced psoriasis mouse model. We then assessed the pathogenic mechanism focusing on immune cells producing IL-1β and IL-17 along with gene expression profiles associated with psoriasis in mouse model and public database on patients. Moreover, therapeutic potential of IL-1β blocking in such context was assessed.

**Results:**

We found that autophagy deficiency in myeloid cells exacerbated neutrophilic inflammation and disease pathogenesis in mice with psoriasis. This autophagy-dependent effect was associated with a significant increase in IL-1β production from myeloid cells, particularly macrophages, *Cxcl2* expression, and IL-17 A producing T cells including γδ T cells. Supporting this, treatment with systemic IL-1 receptor blocking antibody or topical saccharin, a disaccharide suppressing pro-IL-1β expression, led to the alleviation of neutrophilia and psoriatic skin inflammation linked to autophagy deficiency. The pathophysiological relevance of this finding was supported by dysregulation of autophagy-related genes and their correlation with Th17 cytokines in psoriatic skin lesion from patients with psoriasis.

**Conclusions:**

Our results suggest that autophagy dysfunction in myeloid cells, especially macrophages, along with IL-1β dysregulation has a causal role in neutrophilic inflammation and psoriasis pathogenesis.

**Supplementary Information:**

The online version contains supplementary material available at 10.1186/s13578-024-01238-0.

## Background

Psoriasis is an inflammatory skin disease characterized by hyperproliferative epidermal keratinocytes (KC) and excessive infiltration of immune cells, including lymphocytes, neutrophils, and macrophages, into the inflamed skin lesion, leading to the production of various cytokines, such as TNF-α, IL-23, and IL-17 [1–4]. In particular, IL-17, produced by helper T cell 17 (Th17) and γδ T cells, plays a key role in psoriasis pathogenesis by promoting hyperplasia of epidermal KC and the production of chemokines such as CXCL1, CXCL2, CXCL3, CXCL5, and CXCL8 for the recruitment of neutrophils and macrophages into psoriatic lesions [4, 5]. Among others, accumulation of neutrophils in psoriatic plaques is a histopathological hallmark of psoriasis [3, 6, 7]. Neutrophils are considered a key contributor to the pathogenesis of numerous chronic inflammatory and autoimmune diseases [8, 9]. Neutrophils can directly produce IL-17 [10] or indirectly promote the differentiation and proliferation of Th17 cells [11], supporting the notion of neutrophils as the principal cellular mediators in the pathogenesis of psoriasis [7]. Accordingly, therapeutic strategies that target neutrophilic and type 3 inflammation have led to the development of targeted biologics such as antibodies against TNF-α, IL-17, IL-17R, IL-12/23, IL-23 [3, 5], CXCL8 [6], and E-selectin mediating neutrophil recruitment [12]. These therapies have greatly improved the management of patients with moderate to severe psoriasis [13, 14]. However, only a subset of patients achieve the desired level of clinical benefit, such as reaching a psoriasis area severity index (PASI) 100 response (complete clearance of psoriasis lesions) [13, 14]. Thus, there is a growing attention to identify additional key factors that contribute to disease severity and treatment response.

IL-1β is primarily produced by myeloid cells, including macrophages, and is abundantly expressed in psoriatic skin lesions than in healthy skin [1, 15]. IL-1β contributes to neutrophil recruitment by inducing the expression of neutrophilic chemokines such as CXC and CCL chemokines in different cell types including macrophages and KC [16]. In addition, IL-1β induces the release of neutrophil chemokines indirectly by driving the development of IL-17-producing pathogenic Th17 and γδ T cells [17–21]. IL-23 has been established as a key player in the pathogenesis of psoriasis by promoting the generation of pathogenic Th17 and IL-17-producing γδ T cells [1, 2, 4], which can be synergized upon combination with IL-1β [17–21]. Consistent with this finding, IL-1β levels in the plasma and skin of patients with psoriasis show a positive correlation with PASI score [22]. Furthermore, a genetic study has shown an association between *IL1B* polymorphisms and late onset of psoriasis [23]. Despite the significance of IL-1β in the pathogenesis of psoriasis, the mechanisms underlying IL-1β dysregulation and rational therapeutic modulation in psoriasis remain to be elucidated.

Autophagy is a cellular process that maintains metabolic balance, performs essential quality control on organelles, and plays a pleiotropic role in controlling immune responses and inflammation [24–26]. Dysfunctional autophagy has been associated with diverse disorders [27, 28]. In particular, autophagy protects against several inflammatory diseases, such as chronic rhinosinusitis (CRS) [29], gut inflammation in inflammatory bowel disease (IBD) [30], atherosclerotic progression [31], and liver injury [32], by regulating IL-1β production through degradation of the inflammasome components ASC and NLRP3 [33–35]. Moreover, genetic polymorphisms in the autophagy gene *ATG16L1* contribute to the risk of psoriasis [36], and impaired autophagy due to lysosomal dysfunction is observed in chronic atopic dermatitis and psoriasis lesions [37]. However, the effect of autophagy on psoriatic skin inflammation appears to be complex and context-dependent. Enhancing autophagy by cis-khellactone treatment alleviates imiquimod (IMQ)-induced psoriasis by inhibiting pro-inflammatory macrophages, suggesting an anti-inflammatory role of autophagy in psoriasis [38]. In contrast, autophagy deficiency in KC attenuates IMQ-induced skin inflammation by inhibiting HMGB1 secretion [39]. This pro-inflammatory role of autophagy is supported by another study indicating that autophagy is required for neutrophil-mediated inflammation [40]. In this regard, the contribution of autophagy to the pathogenesis of psoriasis in relation to neutrophilic inflammation remains unclear and requires validation for targeting autophagy in psoriasis treatment. In the present study, we sought to assess the direct role of autophagy in myeloid cells with a focus on macrophages in psoriasis development. The results revealed that autophagy deficiency in myeloid cells is causally linked to IL-1β dysregulation and neutrophilic inflammation in a murine model of psoriasis, which was supported by the analysis of gene expression profiles in psoriatic skin lesions from patients.

## Methods

### Mouse

All mouse experiments were conducted in accordance with the Institutional Animal Care and Use Committee (IACUC) of Asan Institute for Life Sciences. *Atg7*^fl/fl^;LysM-Cre mice with myeloid cell-specific deletion of *Atg7* were generated by crossing LysM-Cre mice (Jackson Laboratories, stock number 4781) with *Atg7*^fl/fl^ mice (kindly provided by Prof. Masaaki Komatsu, Juntendo University, Tokyo, Japan). *Atg7*^fl/fl^ littermate mice were used as control mice. Mice were genotyped with PCR using established primers [29].

### Mouse treatment

Aldara cream containing 5% imiquimod (3 M Health Care Limited, NDC 0089-0610-12, UK) was applied daily on the inner side of the mouse ears for 8 days to induce psoriasis-like inflammation [41]. Ear thickness was measured using a digital micrometer (#KM-BMB1-25; Mitutoyo, Kawasaki, Japan) every other day, and ear swelling was calculated as the difference between the thickness on the first day and the thickness on the measured day. To block the IL-1 receptor, an anti-IL-1R1 antibody was intraperitoneally injected into the mice (Clone: JAMA-147, #BE052, RRID: AB_2661843; Bioxcell, Lebanon, NH) every other day for systemic neutralization (12 mg/kg) and intranasally injected on alternate days for local neutralization (2 mg/kg), all 1 h before Aldara cream application. Control mice were treated with DPBS instead of the antibody. In another experiment, saccharin suspended in DPBS (8 mg/kg, 200 µg/200 µL onto each ear) or SB225002 (Sigma; 1 mg/kg, 10 µg/100 µL onto each ear) was administered onto the ear of mice by plastic bandage daily 1 h before Aldara cream application during the experimental period.

### Histology

For histological analysis, the ear tissues of the mice were harvested at the end of the experiment, and fixed with 4% formaldehyde. A pathologist from the Asan Medical Center embedded the tissue in paraffin, sectioned it at 3 μm thickness, and stained the slides with haematoxylin and eosin (H&E). Images were captured on a microscope (DMi1, Leica, Wetzlar, Germany), and epidermal thickness was measured and analysed using the Leica LAS v4 software.

### Severity score of ear skin inflammation

The severity of inflammation of mouse ear skin was scored at the end of the experiment on the basis of clinical PASI, as previously described [41]. Erythema, scaling, and thickness were scored on a scale from 0 to 4 each, and they were depicted as the cumulative score on a scale from 0 to 12 for evaluating the severity of psoriatic skin lesion.

### Flow cytometry

Draining lymph nodes were dissected from mice with psoriatic-like inflammation or control mice. Subsequently, the cells were dissociated and collected via centrifugation for 5 min at 1500 rpm. The cells were stimulated with 50 ng/mL phorbol 12-myristate 13-acetate (PMA) (Sigma-Aldrich, St. Louis, MO) and 500 ng/mL ionomycin (Sigma-Aldrich). Thereafter, GolgiPlug (BD Biosciences) and GolgiStop (BD Biosciences) were added, and the cells were incubated for an additional 3 h (total incubation time 4 h) and stained for surface markers, followed by fixation and permeabilization with Cytofix/Cytoperm solution (BD Pharmingen). The cells were stained to detect IL-17 A and subjected to FACS analysis on Canto2 (BD Biosciences). The following antibodies were used in this study: Live/Dead Fixable Aqua Dead cell stain kit (Invitrogen, L34957, Carlsbad, CA), PerCP Hamster anti-mouse CD3 (BD Pharmingen, 553,067, RRID: AB_394599, San Diego, CA), APC Rat anti-mouse CD4 (BD Pharmingen, 553,051, RRID: AB_398528), FITC Hamster anti-mouse γδTCR (BD Pharmingen, 553,177, RRID: AB_394688), PE Rat anti-mouse CD8 (BD Pharmingen, 553,032, RRID: AB_394571), and PE-Cy7 Rat anti-mouse/rat IL-17 A PE/Cy7 (eBioscience, 25-7177-82, RRID: AB_10732356, San Diego, CA).

### Cytokine measurement using ELISA

For preparing tissue samples for ELISA, psoriasis-induced or control ear tissues dissected from mice were washed, homogenized in chilled PBS, and sonicated to break the cell membrane. Soluble protein extracts were harvested and stored at − 80 °C. For preparing ELISA samples from mouse peritoneal macrophages, 8- to 10-week-old mice were intraperitoneally injected with 3.85% thioglycolate media (211,260, BD Biosciences) [42]. Peritoneal macrophages collected from the peritoneal cavity were seeded in a culture plate and incubated for 3 h. Non-adherent cells were washed away with DMEM, leaving only the adherent cells. These cells were stimulated by R848 treatment (1 µg/mL; tlrl-r848, InvivoGen, San Diego, CA) in the presence or absence of saccharin (indicated concentrations; S1002, Sigma-Aldrich) for 8 h for measuring TNF-α, IL-6, and IL-12p40 or for 4 h followed by ATP addition (5 mM; A6419, Sigma-Aldrich) for IL-1β measurement. After incubation, culture supernatants were harvested and stored at − 80 °C until further use for ELISA. Each cytokine concentration was determined using mouse DuoSet ELISA kits (R&D systems, Minneapolis, MN) for IL-1β (DY401), IL-6 (DY406), TNF-α (DY410), and IL-12p40 (DY499), according to the manufacturer’s instructions. The levels of the indicated cytokines were measured using a luminescence microplate reader (VICTOR X4, Perkin Elmer, Waltham, MA). The concentrations of each cytokine were calculated and analysed on the basis of their respective standard curves.

### RNA isolation & qPCR

For RNA extraction, the ears of the mice were collected and incubated in RNAlater solution (AM7020, Invitrogen, Waltham, MA). RNA purification and reverse transcription were performed using the RNease Mini Kit (74,134, Qiagen GmbH, Hilden, Germany) and ReverTra Ace qPCR RT kit (FSQ-101, Toyobo, Osaka, Japan), respectively, according to the manufacturer’s instructions. For qRT-PCR, cDNA and primers, along with SYBR Green RT-PCR master mix (QPK-201, Toyobo), were mixed in a 96-well PCR plate (Roche Diagnostics, Basel, Switzerland). The PCR product was analysed using the LightCycler 480 RT-PCR system (Roche Diagnostics). The following primers were used in this study: 5′-GAA TGA CCT GTT CTT TGA AGT-3′ (forward) and 5′-TTT GTT CAT CTC GGA GCC-3′ (reverse) for mouse *Il1b*; 5′-TGG AGT CAC AGA AGG AGT GGC TAA G-3′ (forward) and 5′-TCT GAC CAC AGT GAG GAA TGT CCA C-3′ (reverse) for mouse *Il6*; 5′-CAG AAG CTA ACC ATC TCC TGG TTT G-3′ (forward) and 5′-TCC GGA GTA ATT TGG TGC TTC ACA C-3′ (reverse) for mouse *Il12p40*; 5′-CCT GTA GCC CAC GTC GTA GC-3′ (forward) and 5′-TTG ACC TCA GCG CTG AGT TG-3′ (reverse) for mouse *Tnfa*; 5′-CCA ACC ACC AGG CTA CAG G-3′ (forward) and 5′-GCG TCA CAC TCA AGC TCT G-3′ (reverse) for mouse *Cxcl2*; 5′-ATG CCC TCT ATT CTG CCA GAT-3′ (forward) and 5′-GTG CTC CGG TTG TAT AAG ATG AC-3′ (reverse) for mouse *Cxcr2*; 5′-TTC AGA TGG GCA TGA ATG TTT CT-3′ (forward) and 5′-CCA AAT CCG AGC TGT TGT TCT AT-3′ (reverse) for mouse *Il23*; 5′-GCA ACG GGA AGA TTC TGA AG-3′ (forward) and 5′- TGACAA ACT TCT GCC TGACG-3′ (reverse) for mouse *Il1a*; 5′-TGG AGA CCT GGA ATC AGA CAA C-3′ (forward) and 5′-TAT TTT CAG GTG GAT CCA TTT C-3′ (reverse) for mouse *Il18*; 5′-TCC AAC TCC AAG ATT TCC CCG-3′ (forward) and 5′-CAT GCA GTA GAC ATG GCA GAA-3′ (reverse) for mouse *Il33*; 5′-CTG TGC CTT GGT AGC ATC TAT G-3′ (forward) and 5′-GCA GAG TCT CGC CAT TAT GAT TC-3′ (reverse) for mouse *Il12p35*; 5′-TAA TGG TGG ACC GCA ACA ACG-3′ (forward) and 5′-GAC GGA ATA CAG GGC TT CG-3′ (reverse) for mouse *Tgfb1*; 5′-CCA CAG CCC TCT CCA TCA ACT ATA AGC-3′ (forward) and 5′-AGC TCT TCA ACT GGA GAG CAG TTG AGG-3′ (reverse) for mouse *Ifnb*; 5′-ACA GCA AGG CGA AAA AGG ATG-3′ (forward) and 5′-TGG TGG ACC ACT CGG ATG A-3′ (reverse) for mouse *Ifng*; 5′-CAT CGG CAT TTT GAA CGA GGT CA-3′ (forward) and 5′-CTT ATC GAT GAA TCC AGG CAT CG-3′ (reverse) for mouse *Il4*; 5′-GAA AGA GAC CTT GAC ACA GCT G-3′ (forward) and 5′-GAA CTC TTG CAG GTA ATC CAG G-3′ (reverse) for mouse *Il5*; 5′-AGA CCA GAC TCC CCT GTG CA-3′ (forward) and 5′-TGG GTC CTG TAG ATG GCA TTG-3′ (reverse) for mouse *Il13*; 5′-CAA GGC TGG TCC ATG CTC C-3′ (forward) and 5′-TGC TAT CAC TTC CTT TCT GTT GC -3′ (reverse) for mouse *Cxcl1*; 5′-CCC AAT GAG TAG GCT GGA GA-3′ (forward) and 5′-TCT GGA CCC ATT CCT TCT TG -3′ (reverse) for mouse *Ccl2*; 5′-AGA TCT CTG CAG CTG CCC TCA-3′ (forward) and 5′-GGA GCA CTT GCT GCT GGT GTA G-3′ (reverse) for mouse *Ccl5*; 5′-TGG GAA GAC AGC GTT GGA G-3′ (forward) and 5′-AGG CGA GGT GCT TGA TGT G-3′ (reverse) for mouse *Hpgds*; 5′-CTG CTG GTC ATC AAG ATG TAC G-3′ (forward) and 5′-CCC AGG TAG GCC ACG TGT GT-3′ (reverse) for mouse *Ptges* (mPGES-1); 5′-GCA TTC TTT GCC CAG CAC TT-3′ (forward) and 5′-AGA CCA GGC ACC GAC CAA AGA-3′ (reverse) for mouse *Ptgs2*; and 5′-GGC TGT ATT CCC CTC CAT CG-3′ (forward) and 5′-CCA GTT GGT AAC AAT GCC ATG T-3′ (reverse) for mouse *ActB*.

### Immunofluorescence staining

The tissue sections were immunostained with primary antibodies specific for IL-1β (Cell Signaling Technology, 3A6, #12,242; 1:100), CD68 (AbD Serotec, MCA1957, RRID: AB_322219; 1:100), or Ly6G (Bioxcell, 1A8, #BE0075-1, RRID: AB_1107721; 1:100) for 1 h at room temperature followed by overnight incubation at 4 °C. Secondary antibodies that matched the following primary antibodies were incubated for 30 min at room temperature: goat anti-mouse F(ab′)2-Alexa Fluor 488 (Jackson ImmunoResearch, 705-546-147, RRID: AB_2340430; 1:250, west grove, PA) and goat anti-rat F(ab′)2-Alexa Fluor 647 (Jackson ImmunoResearch, 712-606-153, RRID: AB_2340696; 1:250). After DAPI staining, slides treated with ProLong Gold anti-fade mounting reagent (Molecular Probes, P36930, InvivoGen) were mounted with coverslips. Images were captured using an LSM 880 confocal microscope (Carl Zeiss). Images were processed using the ZEN blue software (Carl Zeiss) and analysed using ImageJ (NIH and LOCI).

### GEO analysis

Human gene expression from psoriatic skin biopsies was re-analysed from Gene Expression Omnibus datasets (GSE13355, GSE30999) [43, 44]. Information on patients with psoriasis and skin samples has been provided previously [43, 44]. Statistical comparisons were calculated using the nonparametric Mann–Whitney *U* test or Student’s *t* test, and correlations were calculated using the nonparametric Spearman correlation test.

### Statistics

All data were analysed using the GraphPad Prism software. Statistical comparisons were conducted using the nonparametric Mann–Whitney *U* test. *P* values of 0.05 or less were considered significant.

## Results

### Myeloid cell-specific *Atg7* deficiency exacerbates psoriatic skin inflammation in vivo

**Fig. 1:**
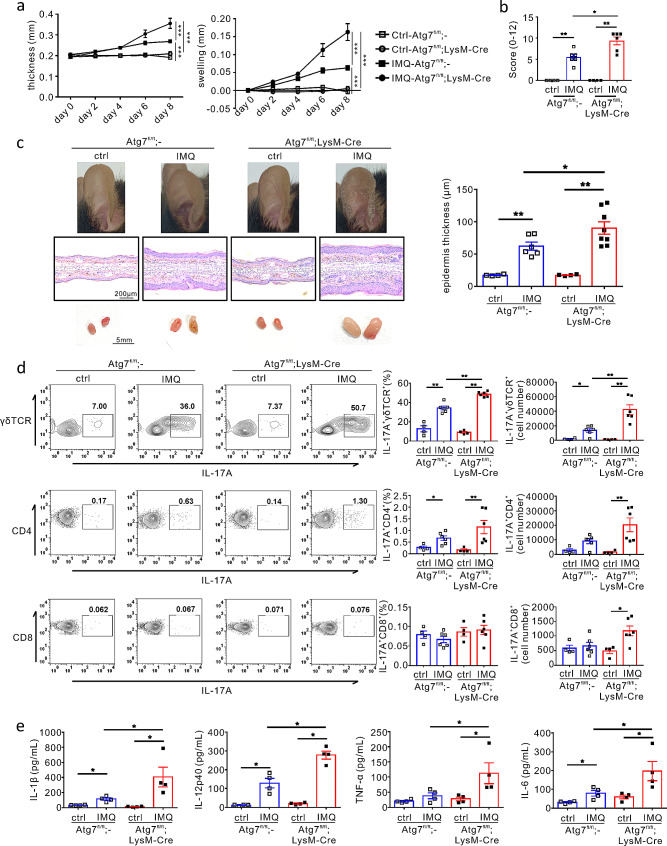
Effect of impaired autophagy in myeloid cells on psoriatic skin inflammation. An IMQ-induced psoriasis model was established in *Atg7*^fl/fl^ and *Atg7*^fl/fl^;LysM-Cre mice. The mice were sacrificed at the end of the experiment, and tissues were harvested for each analysis. (**a**) Ear thickness and swelling were measured every other day (*n* = 6–8 per group) ***P < 0.001, two-way ANOVA. (**b**) Cumulative clinical score (erythema, scaling, and thickness) on a scale from 0 to 12. All bars indicate the mean ± SEM. *P < 0.05 and **P < 0.01, Mann–Whitney U-test. (**c**) Representative ear images (top), H&E-stained histological images (middle), and draining lymph nodes (bottom) from each mouse group. The statistical bar chart shows the epidermal thickness of the ear tissue. All dots indicate the number of samples. All bars indicate the mean ± SEM. *P < 0.05 and **P < 0.01, Mann–Whitney U-test. (**d**) Flow cytometric analysis of the indicated IL-17 A-producing T cells in the draining lymph nodes. The number of samples is indicated by dots. All bars indicate mean ± SEM. *P < 0.05 and **P < 0.01, Mann–Whitney U-test. (**e**) The levels of IL-1β, IL-12p40, IL-6, and TNF-α in harvested ear tissues from each group of mice, as determined using ELISA. All dots indicate the number of samples. All bars indicate mean ± SEM. *P < 0.05 and **P < 0.01, Mann–Whitney U-test

To explore the direct role of autophagy in myeloid cells in the pathogenesis of psoriasis, we developed a model of psoriasis using *Atg7*^fl/fl^;LysM-Cre mice, which selectively exhibit a deficiency of the autophagy gene *Atg7* in myeloid cells such as neutrophils and macrophages [45]. Imiquimod (IMQ) cream was topically applied daily on the ears of the mice over 8 days, and skin inflammation was assessed by measuring the ear thickness every other day. Ear thickness and swelling were significantly increased upon IMQ application in both *Atg7*^fl/fl^;LysM-Cre and *Atg7*^fl/fl^ mice; however, this increase was more significantly pronounced in the *Atg7*^fl/fl^;LysM-Cre mice than in the *Atg7*^fl/fl^ mice (Fig. 1a). Consistent with this result, the *Atg7*^fl/fl^;LysM-Cre mice showed a more severe IMQ-induced PASI score and inflammatory response in the ears than the *Atg7*^fl/fl^ mice (Fig. 1b and c *upper*), along with significantly aggravated epidermis thickness (Fig. 1c, *middle and right*). In addition, IMQ-induced swelling of skin-draining lymph nodes (dLNs) was more pronounced in the *Atg7*^fl/fl^;LysM-Cre mice than in the *Atg7*^fl/fl^ mice (Fig. 1c *bottom*). These results suggest that autophagy in myeloid cells plays a protective role in the pathogenesis of psoriasis.

Considering the critical role of γδ T cells and T helper 17 cells (Th17 cells) in the pathogenesis of psoriasis [21], we assessed the effect of autophagy deficiency in myeloid cells on T cells isolated from dLNs in *Atg7*^fl/fl^;LysM-Cre or *Atg7*^fl/fl^ mice using flow cytometry. γδ T cells are the major cellular source of IL-17 A in the IMQ-induced mouse model of psoriasis [18]. In both groups of mice, IMQ application significantly increased the population of IL-17 A^+^γδ T cells, which was further significantly elevated by autophagy deficiency, in terms of percentage and absolute numbers (Fig. 1d *upper*). In comparison, IL-17 A^+^CD4^+^ T cells populations in the dLNs were clearly increased in both groups upon IMQ application but were not significantly affected by autophagy deficiency (Fig. 1d *middle*). Similarly, autophagy deficiency exhibited a marginal effect on the population of IL-17 A^+^CD8^+^ T cells in dLNs of IMQ-treated mice, with a significant effect only on the absolute number (Fig. 1d *bottom*).

On the basis of the aforementioned findings regarding the association of autophagy deficiency in myeloid cells with exacerbated skin inflammation and increased populations of IL-17 A-producing T cells, especially γδ T cells, we sought to understand the molecular mechanism underlying this effect. To this end, we examined the production of pro-inflammatory cytokines, which play a crucial role in the induction and differentiation of IL-17 A^+^γδ T cells and Th17 cells [1, 2]. We observed a significant increase in the production of IL-1β, IL-12p40, and IL-6 in the IMQ-treated ears of mice compared with that in untreated mice, whereas the TNF-α level was only significantly elevated in IMQ-treated *Atg7*^fl/fl^;LysM-Cre mice (Fig. 1e). Notably, all these cytokines further increased in the *Atg7*^fl/fl^;LysM-Cre mice compared with those in *Atg7*^fl/fl^ mice (Fig. 1e), indicating that autophagy loss in myeloid cells promoted pro-inflammatory cytokine production, thereby contributing to the increased population of IL-17 A-producing T cells, particularly IL-17 A^+^γδ T cells. Collectively, these results suggest a protective role of autophagy in myeloid cells in the pathogenesis of skin inflammation in the IMQ-induced mouse model of psoriasis.

### IL-1β dysregulation in autophagy-deficient macrophages is associated with neutrophilic inflammation

**Fig. 2:**
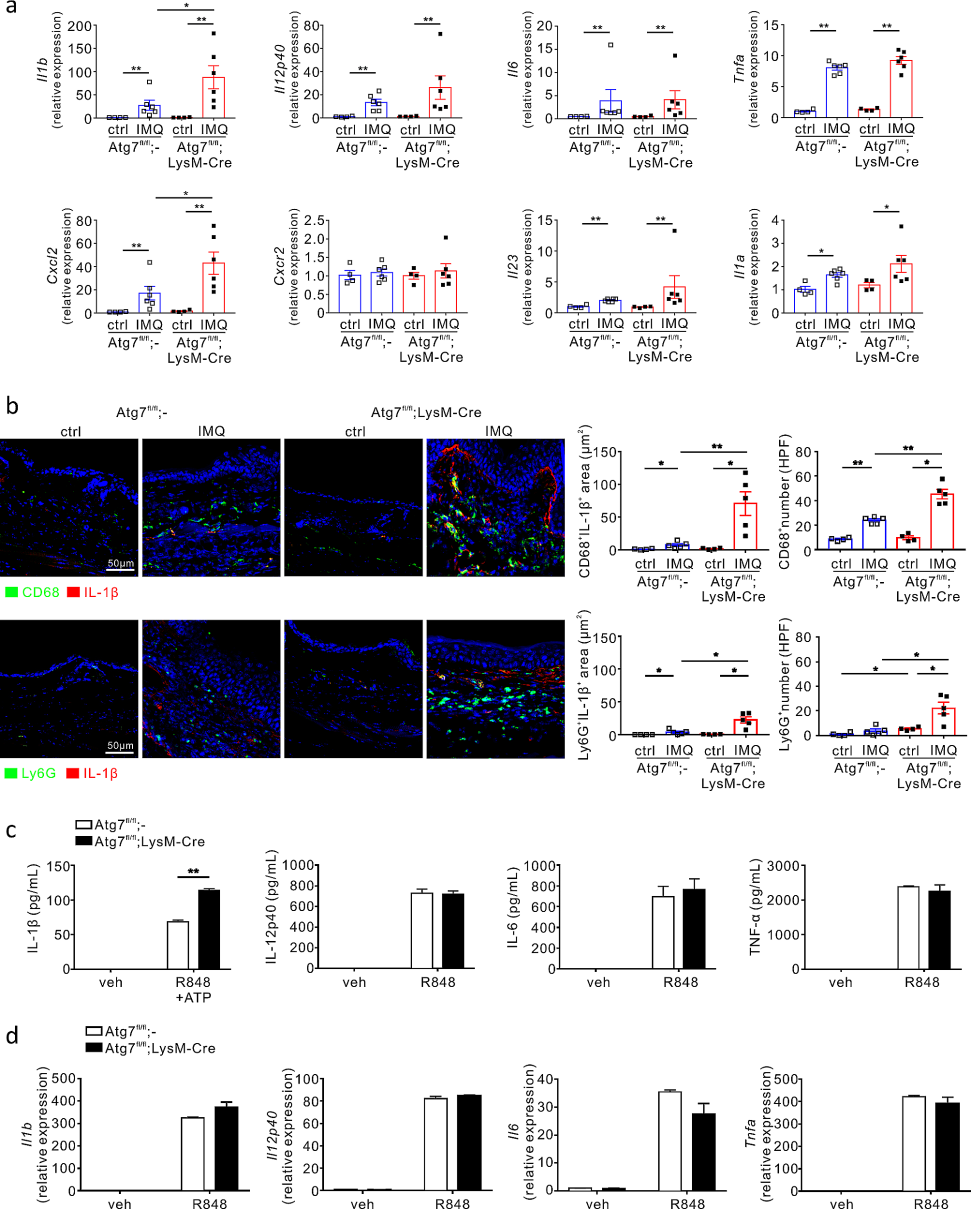
Association of myeloid autophagy with IL-1β expression and neutrophilic inflammation. (**a**) Effect of autophagy deficiency in myeloid cells on the mRNA expression of cytokines related to psoriasis pathogenesis. Relative mRNA levels corresponding to the indicated proteins, as determined using qRT-PCR and normalized to β-actin. All dots indicate the number of samples. All bars indicate the mean ± SEM. *P < 0.05 and **P < 0.01, Mann–Whitney U-test. (**b**) Representative immunostaining for IL-1β, CD68, or Ly6G in the ear tissue from each mouse group. The bar charts show the IL-1β-positive area colocalized with each cell marker and the number of cells for each cell type. Scale bars = 50 μm. All dots indicate the number of samples. All bars indicate the mean ± SEM. *P < 0.05 and **P < 0.01, Mann–Whitney U-test. (**c, d**) Effect of impaired autophagy on the levels of IL-1β, IL-12p40, IL-6, and TNF-α in R848-stimulated peritoneal macrophages from *Atg7*^fl/fl^ or *Atg7*^fl/fl^;LysM-Cre mice, as determined using ELISA (**c**) and qRT-PCR (**d**). Data are representative of at least three independent experiments conducted with duplicates. All bars indicate the mean ± SD. **P < 0.01, Student’s t-test

To further investigate how autophagy exacerbates IMQ-induced psoriatic skin inflammation, we assessed the mRNA expression levels of various cytokines and chemokines in the ears of IMQ-treated mice. Similar to the protein level, the mRNA expression of *Il1b* was significantly increased following IMQ treatment, and this increase was further augmented by myeloid cell-specific autophagy deficiency (Fig. 2a). In comparison, IMQ treatment significantly increased the mRNA expression of *Il12p40*, *Il6*, and *Tnfa* in the mouse ears; however, autophagy deficiency did not increase the expression further (Fig. 2a). Notably, the mRNA expression of *Cxcl2*, a critical chemokine for neutrophil recruitment into inflamed tissue [5], was significantly elevated by IMQ treatment and was further increased by myeloid cell-specific autophagy deficiency (Fig. 2a). In comparison, no significant difference was observed in the mRNA expression of *Cxcl1*, another chemokine for neutrophil recruitment (Supple. Figure 1), and *Cxcr2*, encoding the cognate receptor for CXCL2 (Fig. 2a). In addition, autophagy deficiency did not affect the mRNA expression of *Ccl2* for macrophage recruitment or that of *Ccl5* for the recruitment of leukocytes, such as T cells, eosinophils, basophils, monocytes, NK cells, dendritic cells, and mast cells (Suppl. Figure 1). IMQ treatment significantly elevated the gene expression of *Il23* and *Il1a*, which are critical cytokines for the induction and differentiation of IL-17 A^+^γδ T cells and Th17 cells [1, 2]; however, autophagy deficiency did not further increase their expression (Fig. 2a). Additionally, we observed no significant effect of autophagy deficiency on the expression of diverse cytokines, including IL-1 family cytokines (*Il18* and *Il33*), Th17-cell-inducing TGF-β1 (*Tgfb1*), pro-inflammatory cytokines (*Il12p35* and *Ifnb*), Th1 cytokine (*Ifng*), and Th2 cytokine (*Il4*, *Il5*, and *Il13*) (Supple. Figure 1). In addition, despite the involvement of prostaglandins in pathogenic Th17 cell-driven inflammation including psoriasis [46], impaired autophagy did not affect the expression levels of *Pgts2*, encoding COX-2; *mPges-1* (a prostaglandin E_2_ synthase); and *Hpgds* (a prostaglandin D_2_ synthase) in myeloid cells (Supple. Figure 1). Thus, these results raise the possible involvement of IL-1β and CXCL2 dysregulation, induced by autophagy deficiency in myeloid cells, in promoting neutrophilic inflammation. In support, treatment with an antagonist of CXCR2 (SB225002) that blocks the effect of CXCR2 ligand CXCL2 for neutrophil recruitment significantly alleviated ear thickness and swelling in *Atg7*^fl/fl^ psoriatic mice and, notably, in *Atg7*^fl/fl^;LysM-Cre psoriatic mice with myeloid autophagy deficiency (Supple. Figure 2a). This therapeutic efficacy was further validated by a significant decrease in the PASI score, epidermis thickness and swelling of dLNs (Supple. Figure 2b and c).

Autophagy regulates IL-1β release by degrading inflammasome components such that the loss of autophagy leads to uncontrolled IL-1β production [33–35]. In addition, the IL-1β/CXCL2 axis promotes neutrophil recruitment [16]. Thus, we hypothesized that dysregulation of IL-1β production by impaired myeloid autophagy primarily contributes to the recruitment of myeloid cells, including neutrophils, into the inflamed skin lesion. To this end, we first examined the localization of IL-1β in association with surface markers for macrophages and neutrophils in the ears of psoriasis-induced mice. Upon IMQ treatment, IL-1β production was apparently increased from CD68^+^ macrophages and, to a lesser extent, from Ly6G^+^ neutrophils and was significantly upregulated by autophagy deficiency (Fig. 2b). The dominant colocalization of IL-1β in CD68^+^ macrophages rather than Ly6G^+^ neutrophils suggests that macrophages were the predominant producers of IL-1β linked to autophagy deficiency in our mouse model of psoriasis, consistent with the previous finding that activated monocytes/macrophages are a principal source of IL-1β [47]. Furthermore, the numbers of CD68^+^ macrophages and Ly6G^+^ neutrophils in the skin lesions were markedly increased by autophagy deficiency (Fig. 2b), suggesting that myeloid autophagy coordinates both IL-1β production and neutrophilic inflammation.

We examined the direct contribution of macrophage autophagy to the production of inflammatory cytokines such as IL-1β, IL-12p40, IL-6, and TNF-α in vitro. To this end, peritoneal macrophages were isolated and stimulated with R848, a ligand for Toll-like receptors (TLR) 7 and 8. Subsequently, the production of these cytokines in the culture supernatant was measured using ELISA. In activated macrophages, the production of IL-1β, but not that of IL-12p40, IL-6, and TNF-α, was significantly elevated by autophagy deficiency (Fig. 2c). In comparison, the mRNA expression of these cytokines, including *Il1b*, was not affected by autophagy deficiency in activated macrophages (Fig. 2d), consistent with prior studies on autophagy-mediated targeted pro-IL-1β degradation for IL-1β secretion [33–35]. Collectively, our findings suggest that IL-1β dysregulation in autophagy-deficient myeloid cells, particularly macrophages, is a potential mechanism underlying aggravated neutrophilic inflammation in psoriasis.

### Autophagy deficiency-mediated aggravation of psoriasis is IL-1 dependent

**Fig. 3:**
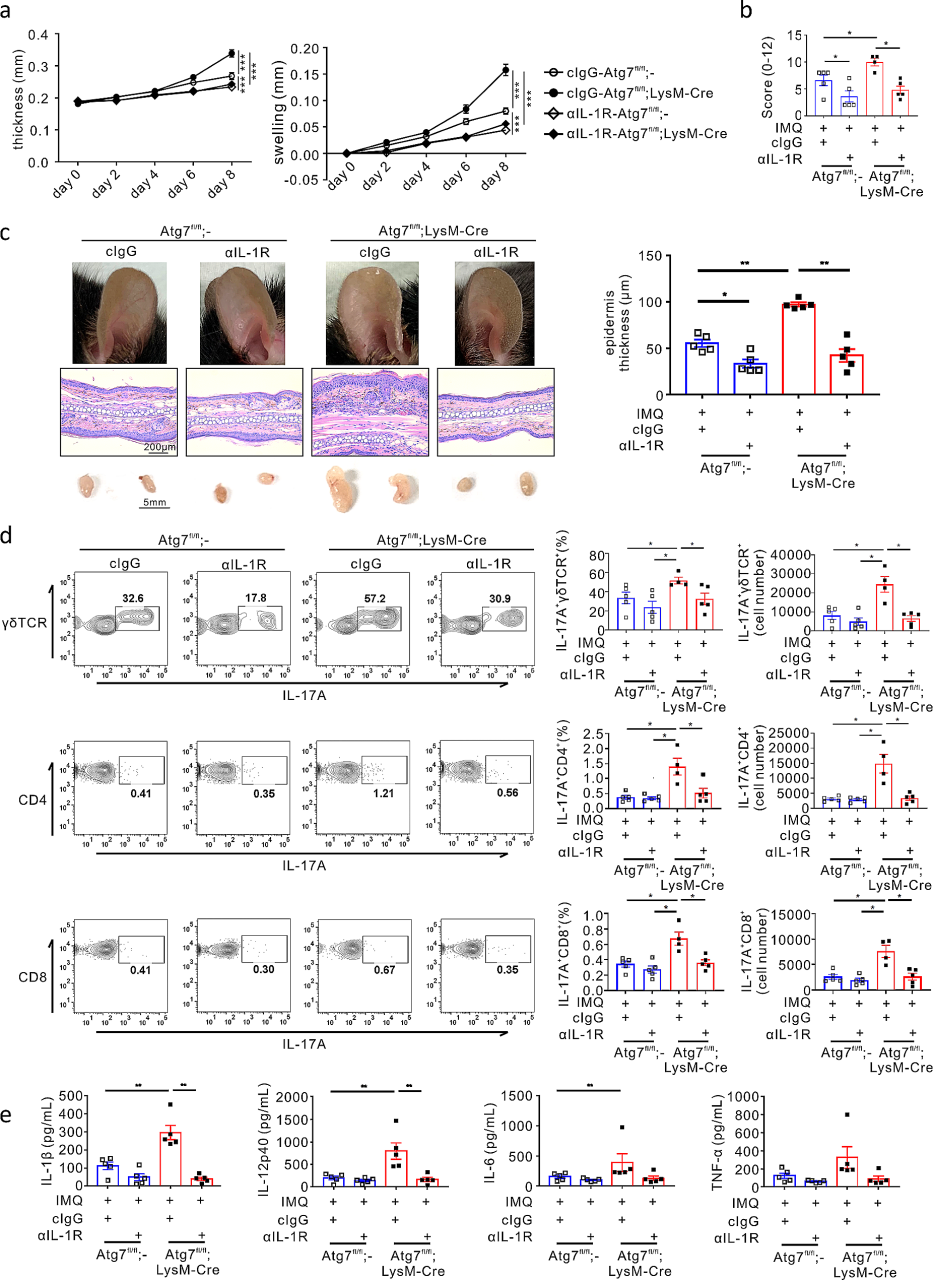
Effect of IL-1-receptor blockade on psoriatic skin inflammation. *Atg7*^fl/fl^ or *Atg7*^fl/fl^;LysM-Cre mice were injected with anti-IL-1R1 antibody or control IgG and sacrificed at the end of the experiment. The tissues were harvested for each analysis. (**a**) Ear thickness and swelling were measured every other day (*n* = 8–10 per group) ***P < 0.001, two-way ANOVA. (**b**) Cumulative clinical score (erythema, scaling, and thickness) on a scale from 0 to 12. All bars indicate the mean ± SEM. *P < 0.05, Mann–Whitney U-test. (**c**) Representative ear images (top), H&E-stained histological images (middle), and draining lymph nodes (bottom) from each mouse group. The bar chart shows the epidermal thickness of ear tissue. All dots indicate the number of samples. All bars indicate the mean ± SEM. *P < 0.05 and **P < 0.01, Mann–Whitney U-test. (**d**) Flow cytometric analysis of the indicated IL-17 A-producing T cells in the draining lymph nodes. All dots indicate the number of samples. All bars indicate the mean ± SEM. *P < 0.05 and **P < 0.01, Mann–Whitney U-test. (**e**) The levels of IL-1β, IL-12p40, IL-6, and TNF-α were determined using ELISA in harvested ear tissues from each mouse group. All dots indicate the number of samples. All bars indicate the mean ± SEM. *P < 0.05 and **P < 0.01, Mann–Whitney U-test

A recent study highlighted the pivotal role of the IL-1β–IL-1R signalling pathway in psoriasis pathogenesis [20]. Thus, to probe the pathogenic role of IL-1β dysregulation in psoriasis, we assessed the effect of IL-1 blockade on psoriatic skin inflammation linked to autophagy deficiency. Mice were systemically administered anti-IL-1R1 antibody, which blocks the interaction of IL-1β with its cognate receptor IL-1R during the development of psoriasis. IL-1 receptor blockade significantly alleviated ear thickness and swelling in both *Atg7*^fl/fl^;LysM-Cre and *Atg7*^fl/fl^ psoriatic mice, regardless of autophagy deficiency (Fig. 3a). Notably, this therapeutic efficacy was most evident in *Atg7*^fl/fl^;LysM-Cre mice, which exhibited an abrogation of pathogenic effect linked to autophagy deficiency (Fig. 3a and b). Similarly, epidermis thickness and swelling of dLNs were significantly decreased in both mouse groups, with the most significant effect in the *Atg7*^fl/fl^;LysM-Cre mice (Fig. 3c). As a control experiment, treatment with anti-IL-1R1 antibody did not affect ear thickness and swelling in both *Atg7*^fl/fl^;LysM-Cre and *Atg7*^fl/fl^ mice without IMQ treatment (data not shown).

We investigated the effects of IL-1 blockade on IL-17 A-producing T cells. Following IMQ treatment, both mouse groups showed an increase in IL-17 A^+^γδ T cell population, which was further increased by autophagy deficiency (Fig. 3d *upper*). Systemic blockade of IL-1 receptor significantly reduced the IL-17 A^+^γδ T cell population in terms of percentages and numbers, particularly in autophagy deficiency. Similarly, the same treatment significantly reduced the percentages and numbers of IL-17 A^+^CD4^+^ T cells and IL-17 A^+^CD8^+^ T cells in dLNs from the *Atg7*^fl/fl^;LysM-Cre mice (Fig. 3d *middle and bottom*). These results suggest a key role of IL-1R signalling in the pathogenesis of psoriasis, linked to autophagy deficiency through its significant effect on IL-17-producing T cells.

We assessed the effect of IL-1 receptor blockade on the production of inflammatory cytokines promoting psoriasis pathogenesis in the ears of IMQ-treated mice. In support of the key role of IL-1β in IMQ-induced psoriasis, blockade of IL-1 receptor significantly diminished the production of IL-12p40 as well as IL-1β in psoriasis-induced skin lesions (Fig. 3e). Although not significant, the production of IL-6 and TNF-α was also apparently reduced by IL-1R blockade in IMQ-treated *Atg7*^fl/fl^;LysM-Cre mice (Fig. 3e). These results suggest that IL-1β dysregulation linked to myeloid autophagy dysfunction can induce psoriatic skin inflammation.

### Autophagy deficiency-mediated neutrophilic inflammation is IL-1 dependent

**Fig. 4:**
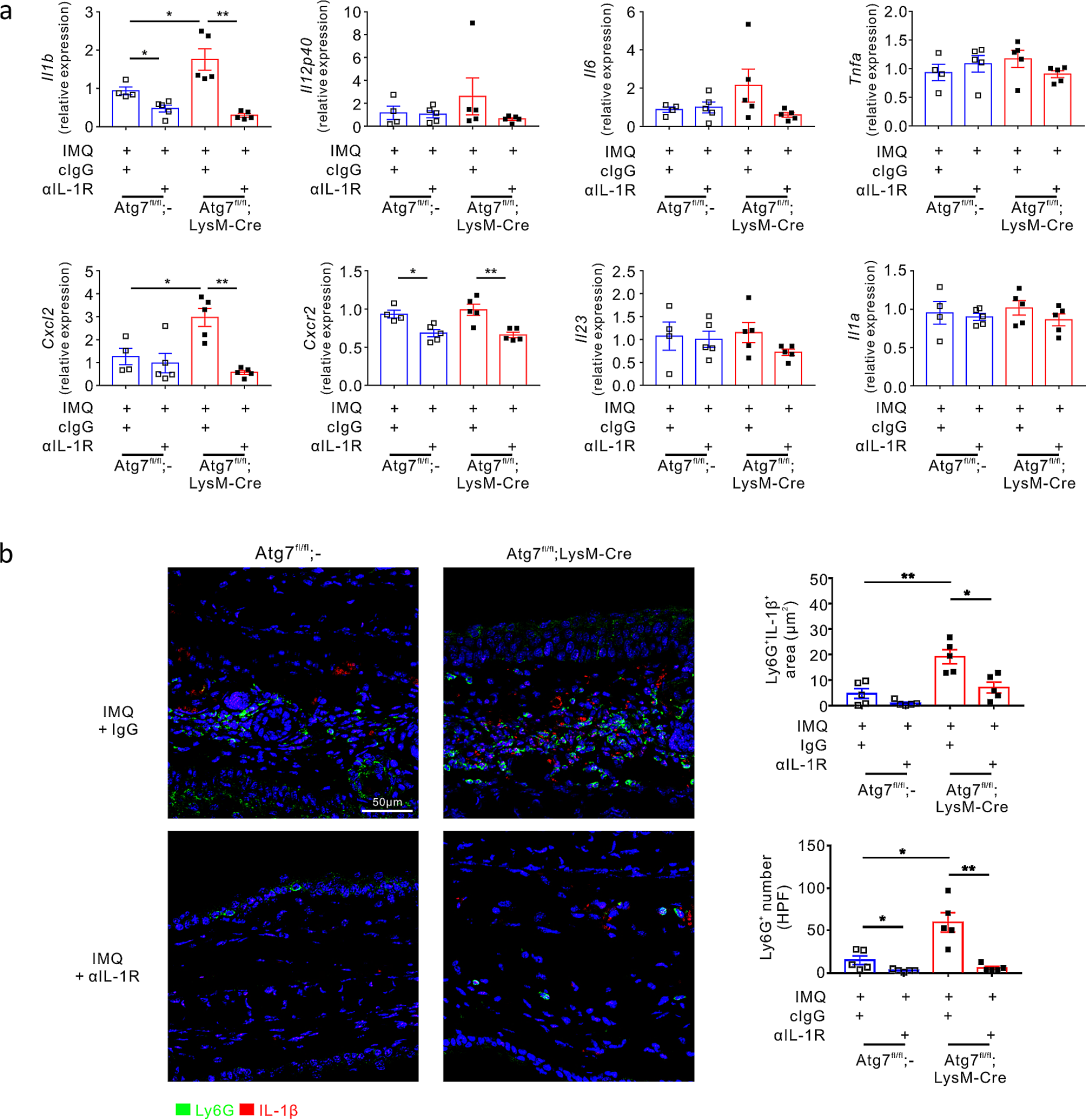
Neutrophilic inflammation in autophagy-deficient psoriatic mice is IL-1-dependent. (**a**) Effect of IL-1R1 signalling blockade on the mRNA expression of genes related to psoriasis pathogenesis. Relative mRNA levels corresponding to the indicated proteins, as determined using qRT-PCR and normalized to β-actin. All dots indicate the number of samples. All bars represent the mean ± SEM. *P < 0.05 and **P < 0.01, as determined using the Mann–Whitney U-test. (**b**) Representative immunostaining for IL-1β and Ly6G in the ear tissue from each mouse group. The bar chart shows the area of IL-1β positivity colocalized with the neutrophil marker and the number of neutrophils. Scale bars = 50 μm. The number of samples is indicated by dots. All bars indicate the mean ± SEM. *P < 0.05 and **P < 0.01, as determined using the Mann–Whitney U-test

To further investigate the therapeutic mechanism of IL-1 receptor blockade, we assessed the mRNA expression of key cytokines and chemokines in the ears of psoriasis-induced mice. We observed a significant upregulation of the mRNA expression of *Il1b* and *Cxcl2* by autophagy deficiency, as expected, which was abrogated by IL-1 receptor blockade (Fig. 4a). In addition, the mRNA expression of *Cxcr2* was significantly reduced. In comparison, no significant changes were observed in the mRNA expression of *Il12p40*, *Il6*, *Tnfa*, *Il23*, and *Il1a*, following IL-1 receptor blockade.

We investigated the recruitment of neutrophils and their expression of IL-1β in the IMQ-induced murine model of psoriasis in the presence or absence of anti-IL-1R1 antibody injection. Colocalization of the augmented IL-1β staining with Ly6G^+^ neutrophils in autophagy deficiency was significantly reduced by the blockade of IL-1 receptor (Fig. 4b), correlating with the expression of *Il1b* and *Cxcl2*. In addition, the anti-IL-1R1 antibody significantly decreased the number of infiltrated Ly6G^+^ neutrophils in both *Atg7*^fl/fl^ and *Atg7*^fl/fl^;LysM-Cre mice (Fig. 4b), supporting the role of IL-1β–IL-1R signalling in driving neutrophilic inflammation during psoriatic skin inflammation, particularly in the context of autophagy deficiency.

### Saccharin suppresses IL-1β expression and alleviates autophagy-deficiency-linked skin inflammation

**Fig. 5:**
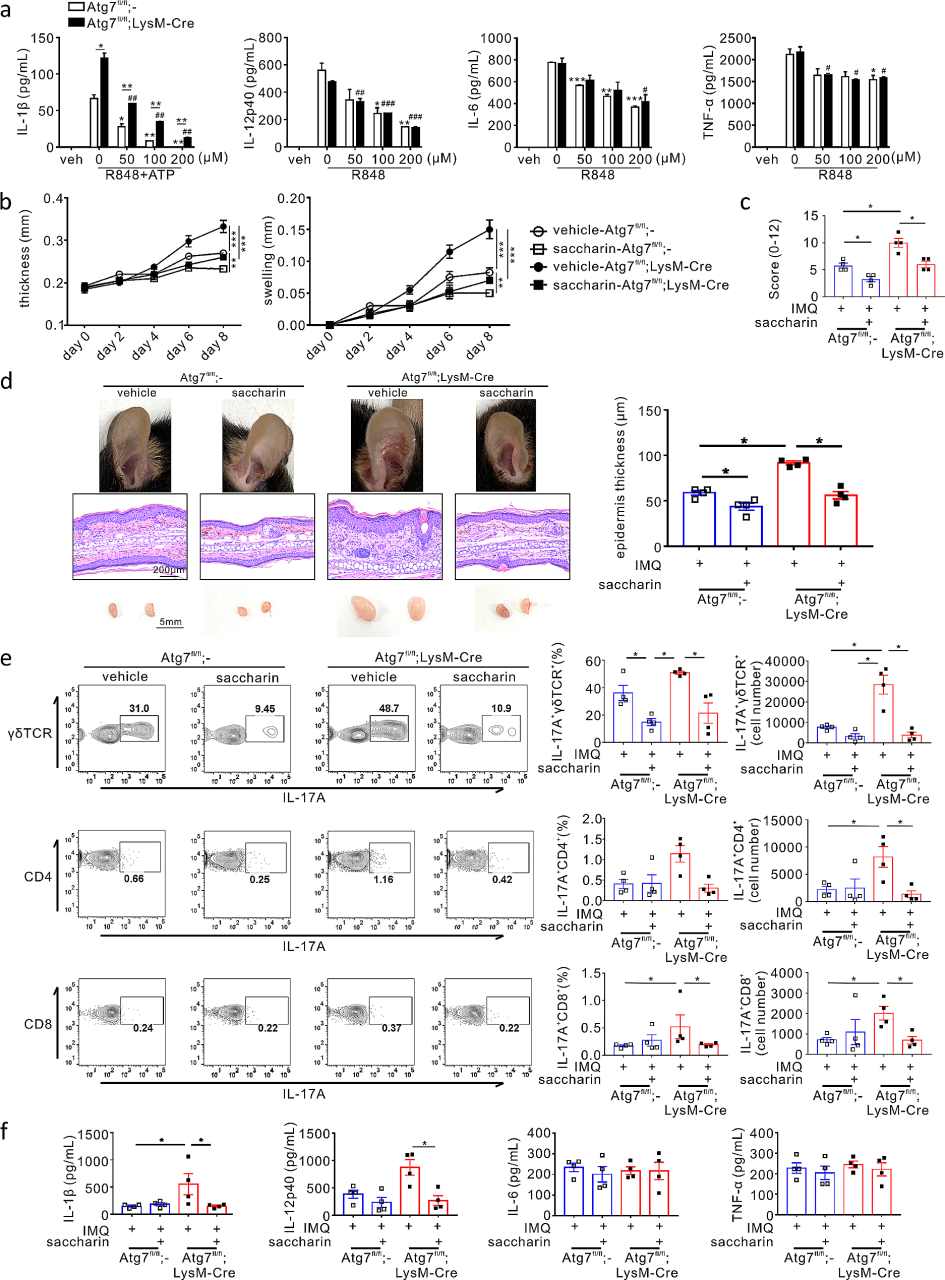
Effect of saccharin on psoriatic skin inflammation. (**a**) Effect of saccharin on the production of IL-1β, IL-12p40, IL-6, and TNF-α from R848-stimulated peritoneal macrophages in *Atg7*^fl/fl^ or *Atg7*^fl/fl^;LysM-Cre mice, as determined using ELISA. Data represent at least two independent experiments conducted with duplicates. All bars indicate the mean ± SD. **P < 0.01, Student’s t-test. (**b-f**) Saccharin or vehicle was applied onto the ear of *Atg7*^fl/fl^ or *Atg7*^fl/fl^;LysM-Cre mice every day prior to IMQ treatment. Mice were sacrificed at the end of the experiment, and tissues were harvested for each analysis. (**b**) Ear thickness and swelling were measured every other day (*n* = 8). ***P < 0.001, two-way ANOVA. (**c**) Cumulative clinical score (erythema, scaling, and thickness) on a scale from 0 to 12. All bars indicate the mean ± SEM. *P < 0.05, Mann–Whitney U-test. (**d**) Representative ear images (top), H&E-stained histological images (middle), and draining lymph nodes (bottom) from each mouse group. The bar chart shows the epidermal thickness of ear tissue. The number of samples is indicated by dots. All bars indicate the mean ± SEM. *P < 0.05 and **P < 0.01, Mann–Whitney U-test. (**e**) Flow cytometric analysis of the indicated IL-17 A-producing T cells in the draining lymph nodes. All dots indicate the number of samples. All bars indicate the mean ± SEM. *P < 0.05 and **P < 0.01, Mann–Whitney U-test. (**f**) The protein levels of IL-1β, IL-12p40, IL-6, and TNF-α in harvested ear tissues from each mouse group, as determined using ELISA. All dots indicate the number of samples. All bars indicate the mean ± SEM. *P < 0.05 and **P < 0.01, Mann–Whitney U-test

Our previous study revealed that artificial sugars, particularly saccharin, exert an anti-inflammatory effect on macrophage IL-1β production in an autophagy-independent manner [48]. Based on this finding, we investigated whether saccharin ameliorates IMQ-induced psoriatic skin inflammation in *Atg7*^fl/fl^;LysM-Cre mice. To this end, we first examined the effects of saccharin on R848-stimulated macrophages in vitro and found that saccharin significantly and dose-dependently reduced the production of IL-1β, in particular, and IL-12p40 by R848-activated macrophages; this effect was comparable between WT and Atg7-deficient macrophages (Fig. 5a). To a lesser extent, the same treatment reduced the production of IL-6 and TNF-α, independent of autophagy deficiency. These results indicated that saccharin exhibits a preferential anti-inflammatory effect on IL-1β and IL-12p40 production, prompting us to examine the therapeutic efficacy of saccharin in psoriasis.

To this end, we induced a murine model of psoriasis in *Atg7*^fl/fl^;LysM-Cre or *Atg7*^fl/fl^ mice in the presence or absence of saccharin treatment. Considering the favourable safety profile of saccharin [49] and easy access to psoriatic skin, saccharin was topically applied to the ears of mice 1 h before IMQ treatment every day for a total of 8 days. Saccharin treatment significantly decreased ear thickness and swelling in *Atg7*^fl/fl^ psoriatic mice and, most notably, in *Atg7*^fl/fl^;LysM-Cre psoriatic mice (Fig. 5b). The PASI scores were also significantly decreased by saccharin treatment in both groups of psoriatic mice (Fig. 5c). This therapeutic efficacy of saccharin was further supported by the assessment of epidermis thickness and swelling of dLNs (Fig. 5d). In control experiment, saccharin treatment alone did not affect ear thickness and swelling in both *Atg7*^fl/fl^;LysM-Cre and *Atg7*^fl/fl^ mice without IMQ treatment (data not shown).

**Fig. 6:**
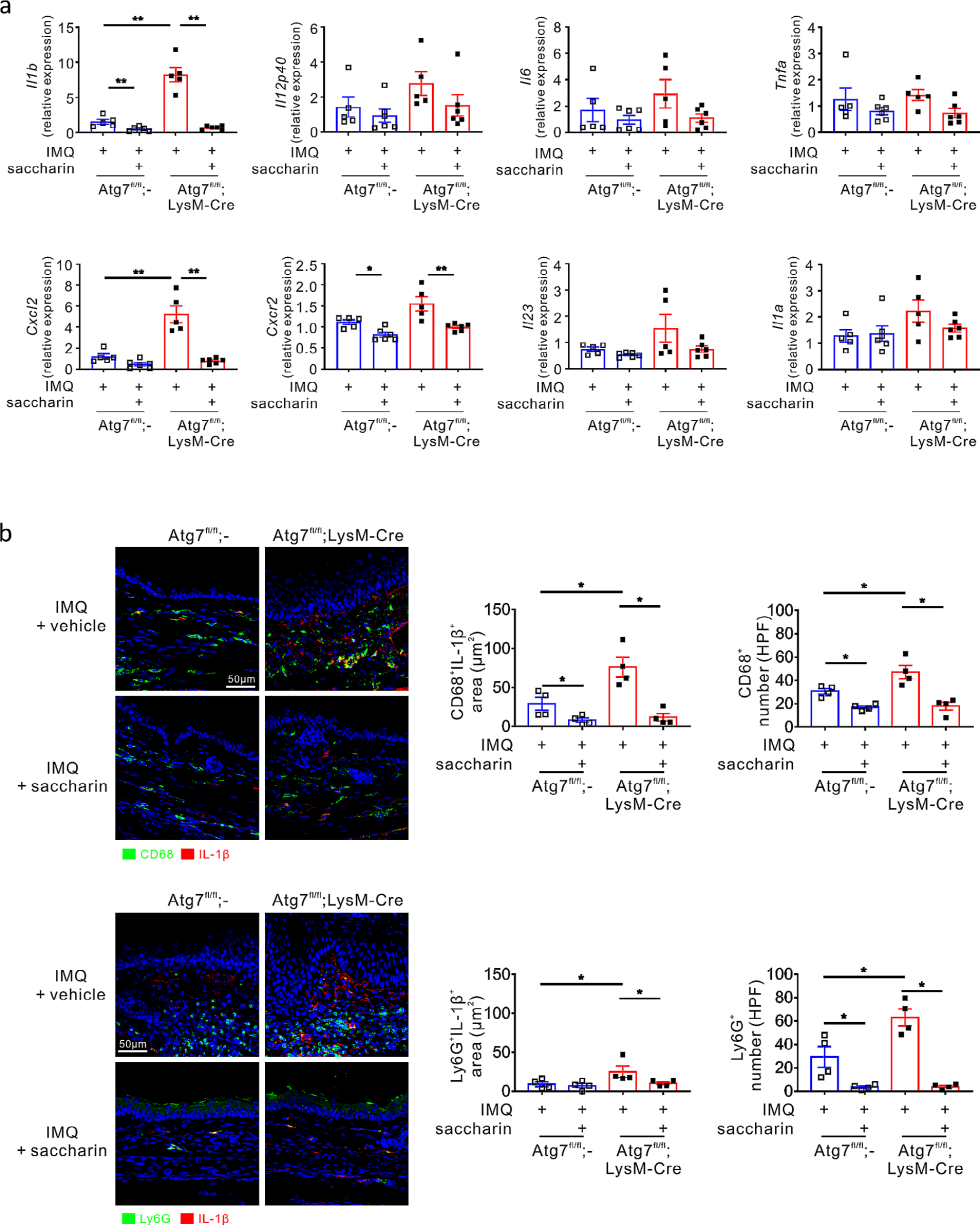
Effect of saccharin on myeloid expression of IL-1β and neutrophilic inflammation. (**a**) Effect of saccharin on the mRNA expression of genes related to psoriasis pathogenesis. Relative mRNA levels corresponding to the indicated proteins, as determined using qRT-PCR and normalized to β-actin. All dots indicate the number of samples. All bars indicate the mean ± SEM. *P < 0.05 and **P < 0.01, Mann–Whitney U-test. (**b**) Representative immunostaining for IL-1β and cell markers CD68 or Ly6G in the ear tissue from each mouse group. The bar chart shows the IL-1β-positive area colocalized with each cell marker and the number of cells for each cell type. Scale bars = 50 μm. All dots indicate the number of samples. All bars indicate the mean ± SEM. *P < 0.05, Mann–Whitney U-test

Next, we analysed the effect of saccharin on IL-17-producing T cells, a key effector in psoriasis. As seen with IL-1 receptor blockade, saccharin treatment, effectively and significantly reduced the percentages and number of IL-17 A^+^γδ T cells, in particular, and IL-17 A^+^CD4^+^ and IL-17 A^+^CD8^+^ cells induced by autophagy deficiency (Fig. 5e). In addition, saccharin treatment significantly decreased the protein levels of IL-1β and IL-12p40 in IMQ-induced *Atg7*^fl/fl^;LysM-Cre mice, which was similar to the effect of IL-1 receptor blockade (Fig. 5f). The same treatment significantly suppressed the augmented mRNA expression levels of *Il1b* and *Cxcl2*, along with *Cxcr2*, in IMQ-induced *Atg7*^fl/fl^;LysM-Cre mice (Fig. 6a). Consistent with the significant effect of saccharin on *Il1b* and *Cxcl2* expression, the infiltration of CD68^+^ macrophages and Ly6G^+^ neutrophils and their production of IL-1β in the psoriatic lesions were markedly reduced by saccharin treatment, particularly in autophagy deficiency (Fig. 6b). Considering that the efficacy of saccharin is comparable to that of IL-1 receptor blockade in reducing the pathological signs of psoriasis, the number of IL-17 A-producing T cells, and inflammatory cytokine levels, we speculate that saccharin is an eligible and alternative candidate for the topical treatment of psoriasis via its anti-inflammatory effect on IL-1β.

### Autophagy-related gene dysregulation and correlation with the Th17 pathway in psoriatic skin lesions

**Fig. 7:**
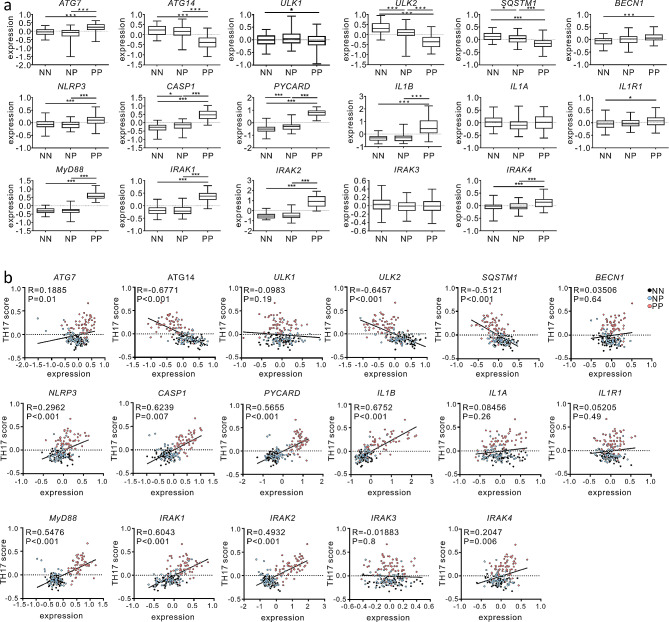
Gene expression analysis in human psoriatic skin biopsy specimens. (**a**) Gene expression profiles were examined in normal, non-lesion skin from healthy donors (NN), non-lesion skin from patients with psoriasis (NP), and psoriatic lesions from patients (PP) using the microarray gene expression dataset (GSE13355). (**b**) Correlation analysis between target genes and TH17 score. The TH17 score was generated by averaging the expression levels of the *IL17A*, *IL17F*, *IL22*, *IL23A*, *IL23R*, and *IL12B* genes. Black, blue, and red dots indicate normal, healthy, non-lesion skin (NN); non-lesion skin from patients with psoriasis (NP); and psoriatic lesions from patients (PP), respectively. *P* values were calculated using Mann–Whitney U-tests (**a**) or the Spearman correlation test (**b**)

To extrapolate whether our findings from the murine model of psoriasis have human relevance, we analysed a public microarray dataset (GSE13355) on gene-expression profiles in skin-biopsy specimens from patients with psoriasis and healthy donor subjects [43]. We first assessed the expression profiles of genes related to autophagy and found that psoriatic skin lesions from patients (PP) showed increased expression of *ATG7* and *BECN1* compared with normal skin biopsies from healthy donors (NN) or normal skin biopsies from patients (NP) (Fig. 7a). In contrast, the expression of other autophagy-related genes, including *ATG14*, *ULK1*, *ULK2*, and *SQSTM1* encoding p62, was significantly decreased in psoriatic skin lesions from patients (PP) compared with that in the other specimens (NN and NP). This reciprocal regulation of autophagy-related genes suggests a potential dysregulation of autophagy in human psoriatic lesions, corroborating a recent report of impaired autophagy due to lysosomal dysfunction in psoriasis lesions from patients [37]. Genes related to inflammasomes (*NLRP3*, *CASP1*, and *PYCARD*) and IL-1β–IL-1R1 signalling (*IL1B*, *IL1R1*, *MyD88*, *IRAK1*, *IRAK2*, and *IRAK4*) were significantly overexpressed in psoriatic skin lesions (Fig. 7a). In contrast, no significant changes were observed in the expression levels of *IL1A* and *IRAK3*, a down-regulator of IL-1β–IL-1R1 signalling (Fig. 7a). Thereafter, we investigated the correlation of Th17-related genes with these genes. Notably, the up-regulated expression of *ATG7* and *BECN1* exhibited moderate positive correlation, whereas the down-regulated genes (*ATG14*, *ULK1*, *ULK2*, and *SQSTM1*) demonstrated a remarkable negative correlation with the Th17 pathway (Fig. 7b). Inflammasome-related genes (*NLRP3* and *PYCARD*) and IL-1β–IL-1R1 signalling-related genes (*IL1B*, *MyD88*, *IRAK1*, *IRAK2*, and *IRAK4*) were significantly and positively correlated with genes related to the Th17 pathway (Fig. 7b). To confirm these results, we analysed an independent microarray dataset (GSE30999) on gene expression profiles in non-lesion or psoriasis lesion samples from patients (Supple. Figure 3) [44]. Similarly, we observed a reciprocal dysregulation of autophagy-related genes (*ATG7* vs. *ATG14* and *SQSTM1*) and overexpression of genes related to inflammasomes (*NLRP3*, *CASP1*, and *PYCARD*) and IL-1β–IL-1R1 signalling (*IL1B*, *IL1R1*, *MyD88*, *IRAK1*, *IRAK2*, and *IRAK4*), which correlated with the Th17 pathway (Supple. Figure 3). Thus, these findings support a potential link between autophagy dysregulation and inflammasome activation for IL-1β production and the inflammatory Th17 pathway in human psoriatic skin inflammation.

## Discussion

**Fig. 8:**
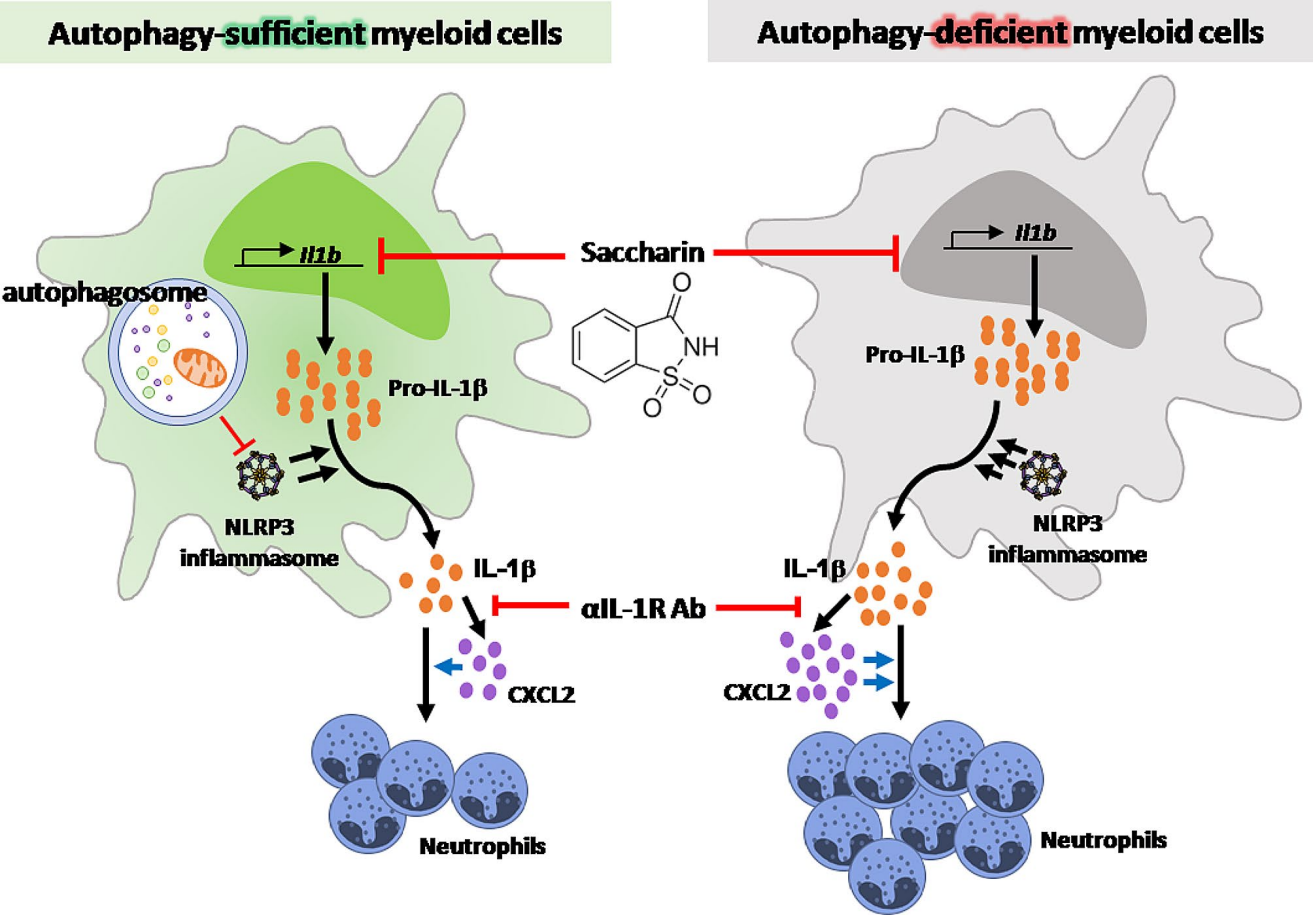
Proposed model of myeloid autophagy deficiency-mediated upregulation of macrophage IL-1β production and exacerbation of neutrophilic inflammation in psoriasis. Autophagy deficiency in myeloid cells aggravated neutrophilic inflammation and severity of psoriasis, such as epidermis thickness and swelling. This aggravation was associated with IL-1β dysregulation in macrophages, *Cxcl2* expression, and IL-17 A producing T cells, which was blocked by treatment with a systemic IL-1 receptor blocking antibody or topical saccharin, a disaccharide suppressing pro-IL-1β expression

Autophagy has been regarded as a regulator of diverse inflammatory diseases, including eosinophilic CRS and IBD, by inducing IL-1β production [29, 30]. Indeed, the accumulation of p62, indicating impaired autophagy, was observed in skin specimens of patients with psoriasis [37]. Recent studies have revealed the crucial role of autophagy in psoriatic skin inflammation, albeit with controversial results regarding the types of cells engaged [38, 39]. Thus, the mechanism underlying the role of autophagy in psoriasis pathogenesis remains unclear and needs to be clarified, along with consideration of autophagy as a promising therapeutic target for treating diverse inflammatory disorders. In this study, we first demonstrated that dysfunctional autophagy in myeloid cells, particularly macrophages, exacerbates psoriatic skin inflammation by inducing IL-1-dependent neutrophilic inflammation. Mechanistically, we showed that the loss of autophagy facilitated uncontrolled release of IL-1β from myeloid cells, which is associated with a high expression of *Cxcl2*, a chemokine responsible for neutrophil recruitment through CXCR2, and with aggravation of neutrophilic inflammation. Furthermore, IL-1R1 blockade was found to alleviate psoriatic skin inflammation, decrease *Cxcl2* expression, and diminish neutrophilia, suggesting that IL-1β dysregulation due to autophagy deficiency plays an important role in psoriasis pathogenesis by driving neutrophilic inflammation (Fig. 8).

To probe the underlying mechanism, we focused on IL-1β dysregulation because impaired autophagy facilitates IL-1β production by suppressing the inflammasome pathway [33–35]. Moreover, IL-1β synergizes with IL-23 to activate IL-17 A-producing γδ T cells, a major contributor to psoriasis [18, 19], and the IL-1β–IL-1R1 signalling pathway plays a crucial role in the pathogenesis of psoriasis [20]. Notably, we observed that the mRNA expression of *Il1b*, but not the expression of other cytokines such as *Il12p40*, *Il6*, *Tnfa*, *Il1a*, and *Il23*, was markedly up-regulated in the ear of psoriatic mice with myeloid autophagy deficiency. In addition, autophagy deficiency led to the prominent production of IL-1β, particularly from macrophages in vivo and isolated macrophages in vitro. Moreover, IL-1R1 blockade significantly reduced the population of IL-17 A-producing γδ T cells, CD4^+^ T cells, and CD8^+^ T cells, consistent with the role of IL-1β in the development of IL-17 A-producing T cells and psoriasis pathogenesis [18–20]. However, we cannot rule out the contribution of IL-1β dysregulation in neutrophils in such context, given a significant upregulation of IL-1β in neutrophils by myeloid autophagy deficiency. In this regard, it requires further study to dissect the role of autophagy deficiency in neutrophils despite the focus of this study on IL-1β dysregulation in macrophages. Collectively, the results of the IL-1 receptor blockade and our present findings suggest that impaired myeloid autophagy promotes psoriatic skin inflammation through an effect on IL-1β dysregulation and consequent IL-17-mediated neutrophilic inflammation, thereby supporting a protective role of myeloid autophagy in the pathogenesis of psoriasis.

To further understand how autophagy regulates neutrophil recruitment into inflamed skin lesions, we assessed the effect of autophagy deficiency on the expression of diverse chemokines implicated in leukocyte recruitment. Among other chemokines tested, we found a significant overexpression of *Cxcl2*, correlating with neutrophil recruitment into the inflamed tissue. This phenomenon was significantly suppressed by IL-1R1 blockade, suggesting that *Cxcl2* expression and neutrophilic inflammation depended on IL-1β dysregulation in our mouse model. This result is supported by reports that IL-1β is involved in CXCL2 production [16] and that loss of IL-1β is related to a selective impairment in CXCL2 production and neutrophil recruitment [50]. Moreover, macrophage-derived CXCL2 controls the early stage of neutrophil recruitment during tissue inflammation [51]. In support, treatment with a CXCR2 antagonist that blocks CXCL2-CXCR2 pathway for neutrophil recruitment significantly alleviated the pathogenesis of psoriasis caused by myeloid autophagy deficiency. In this respect, we speculate that myeloid autophagy could control neutrophilic inflammation in psoriasis through a mechanism involving CXCL2; however, the exact underlying mechanism including other cytokines and chemokines requires further study.

The anti-inflammatory effect of saccharin on psoriatic skin inflammation is pertinent. Saccharin is a safe artificial sweetener over 300 times sweeter than sucrose, with an acceptable daily intake of 5 mg/kg bw/day for human consumption [49]. Saccharin exerts an anti-inflammatory effect by inhibiting iNOS expression in the RAW 264.7 macrophage cell line [52] and reducing IL-1β mRNA expression in the 3T3-L1 adipocyte cell line [53]. Our previous study revealed that saccharin reduces IL-1β production from macrophages via a mechanism dependent on the sweet-taste receptor T1R3 but independent of autophagy and thereby attenuates eosinophilic inflammation in a murine model of ECRS [48]. In diverse inflammatory disorders including psoriasis, the role of autophagy depends on the cell type; therefore, targeting autophagy systemically as a therapeutic modality is challenging [54, 55]. In this regard, saccharin, which exhibits an autophagy-independent anti-inflammatory effect, could be considered a potential candidate for treating psoriasis involving IL-1β dysregulation. IL-1 blockers such as canakinumab (anti-IL-1β antibody) and anakinra (recombinant IL-1R antagonist) are effective against various autoinflammatory diseases including scleritis, Kawasaki disease, osteoarthritis, rheumatoid arthritis, and psoriasis [56–58]. Despite the safety profiles and tolerability of these biologics [56–58], adverse events have been reported, including an increased frequency of non-serious infections of the upper respiratory tract and reactions at the injection site, especially for anakinra [56]. Additionally, canakinumab can trigger severe adverse events such as macrophage activation syndrome [56, 59]. Thus, considering its topical effectiveness and favourable safety profiles, the therapeutic potential of topical saccharin treatment for inflammatory diseases involving IL-1β dysregulation merits further investigation.

Finally, we confirmed the relevance of our findings to humans by analysing biopsy samples from patients with psoriasis. Certain autophagy-related genes (e.g., *ATG7* and *BECN1*) were increased in the psoriatic lesions; however, others such as *ATG14*, *ULK1*, *ULK2*, and *SQSTM1* were significantly down-regulated. Although further studies are required, such decreased expression of *ATG14*, *ULK1*, and *ULK2*, which are required for initiating autophagosome formation [28], may be linked to autophagy dysfunction, consistent with the observation that psoriatic lesions from patients are characterized by impaired autophagy [37, 60]. Furthermore, single nucleotide polymorphisms (SNP) in *ATG16L1* have been linked to the risk of psoriasis [36]. Established therapies for psoriasis, such as retinoids, vitamin D analogues, and UVB phototherapy, can induce autophagy [60]. Considering that autophagy dysregulation facilitates IL-1β release, elevated expression of IL-1β-related genes (*NLRP3*, *CASP1*, *PYCARD*, *IL1B*, *MyD88*, *IRAK1*, *IRAK2*, and *IRAK4*) may cooperate and perpetuate IL-1β dysregulation and associated neutrophilic inflammation. Consistent with this notion, we observed that the expression of Th17 signature genes is inversely correlated with that of autophagy-related genes (*ATG14*, *ULK2*, and *SQSTM1*) but is positively correlated with that of IL-1β-related genes (*NLRP3*, *CASP1*, *PYCARD*, *IL1B*, *MyD88*, *IRAK1*, *IRAK2*, and *IRAK4*). Thus, these results corroborate the involvement of autophagy dysfunction in psoriasis pathogenesis but requires further study to address the clinical relevance of therapeutic modulation of autophagy or IL-1β in psoriasis.

In summary, our study delved into the role of autophagy in myeloid cells with a focus on macrophages and provided an insight into the pathogenic mechanism of psoriasis involving IL-1β dysregulation induced by autophagy deficiency. Our results revealed that myeloid autophagy protects against the development of psoriasis by significantly affecting neutrophilic and type 3 inflammation. Furthermore, our findings imply that enhancing the autophagy pathway specifically in myeloid cells or topical suppression of IL-1β dysregulation, possibly using saccharin, might serve as a safe and potential therapeutic strategy for alleviating neutrophilic inflammation and improving treatment outcomes in patients with psoriasis. However, this study has some limitations. We cannot rule out the contribution of other regulatory mechanisms besides IL-1β and CXCL2 to neutrophilia and psoriasis pathogenesis caused by myeloid autophagy deficiency as we assessed the expression of a selected set of cytokines and chemokines central to inflammatory diseases. It remains unclear whether the findings of this study in IMQ-induced mouse model of psoriasis can be translated to human and merits further study in other mouse models of psoriasis that recapitulate human psoriatic inflammation. Moreover, myeloid autophagy dysregulation needs to be further validated in skin-biopsy specimens from psoriatic patients in addition to the analysis of gene-expression profiles. Nevertheless, our findings provide the direct evidence that myeloid autophagy dysfunction has a causal role in the neutrophilic inflammation of psoriasis and informs potential and affordable therapeutic target for the clinical benefit.

## Conclusion

Our findings link autophagy deficiency in myeloid cells to the aggravated pathogenesis of psoriasis. Impaired autophagy in myeloid cells promoted neutrophil recruitment and aggravated psoriatic skin inflammation through a significant effect on the IL-1β-IL1R1/CXCL2 axis. These findings underscore the context-dependent role of autophagy in the pathogenesis of psoriasis, according to the cell type, and caution against the consideration of autophagy as a therapeutic target in psoriasis. Furthermore, our findings suggest the therapeutic potential of saccharin, a safe disaccharide inhibiting pro-IL-1β expression, for treating psoriasis. Topical treatment with saccharin significantly suppressed the expression of IL-1β and *Cxcl2* and neutrophil recruitment, leading to the alleviation of psoriatic skin inflammation. Considering its anti-inflammatory effects independent of autophagy, saccharin could bypass the requirement for specific therapeutic targeting of autophagy in myeloid cells, and it could be considered a potential candidate for topical psoriasis treatment. In sum, this study proposes a protective role of myeloid autophagy in the pathogenesis of psoriasis via limiting IL-1β-dependent neutrophilic inflammation.

### Electronic supplementary material

Below is the link to the electronic supplementary material.

Supplementary material 1

## Data Availability

All relevant data can be found in the figures and supplementary materials. For any further details required to reanalyze the data presented in this current study, additional information can be obtained from the corresponding author upon reasonable request.

## Abbreviations

Atg7: Autophagy related 7
IMQ: Imiquimod
IL: Interleukin
TNF-α: Tumor necrosis factor alpha
Th17: T helper 17 cell
IL-1R1: Interleukin 1 receptor 1
CXCL: C-X-C motif chemokine ligand
CXCR: C-X-C motif chemokine receptor
