# Supplementary material for: Impaired autophagy in myeloid cells aggravates psoriasis-like skin inflammation through the IL-1β/CXCL2/neutrophil axis
Source: Cell Biosci. 2024 May 4;14:57. doi: 10.1186/s13578-024-01238-0 (PMC11069248; doi:10.1186/s13578-024-01238-0)
Supplement: Supplementary file 1 — Supplementary material 1 [file 13578_2024_1238_MOESM1_ESM.docx]

**Supplementary Figures**


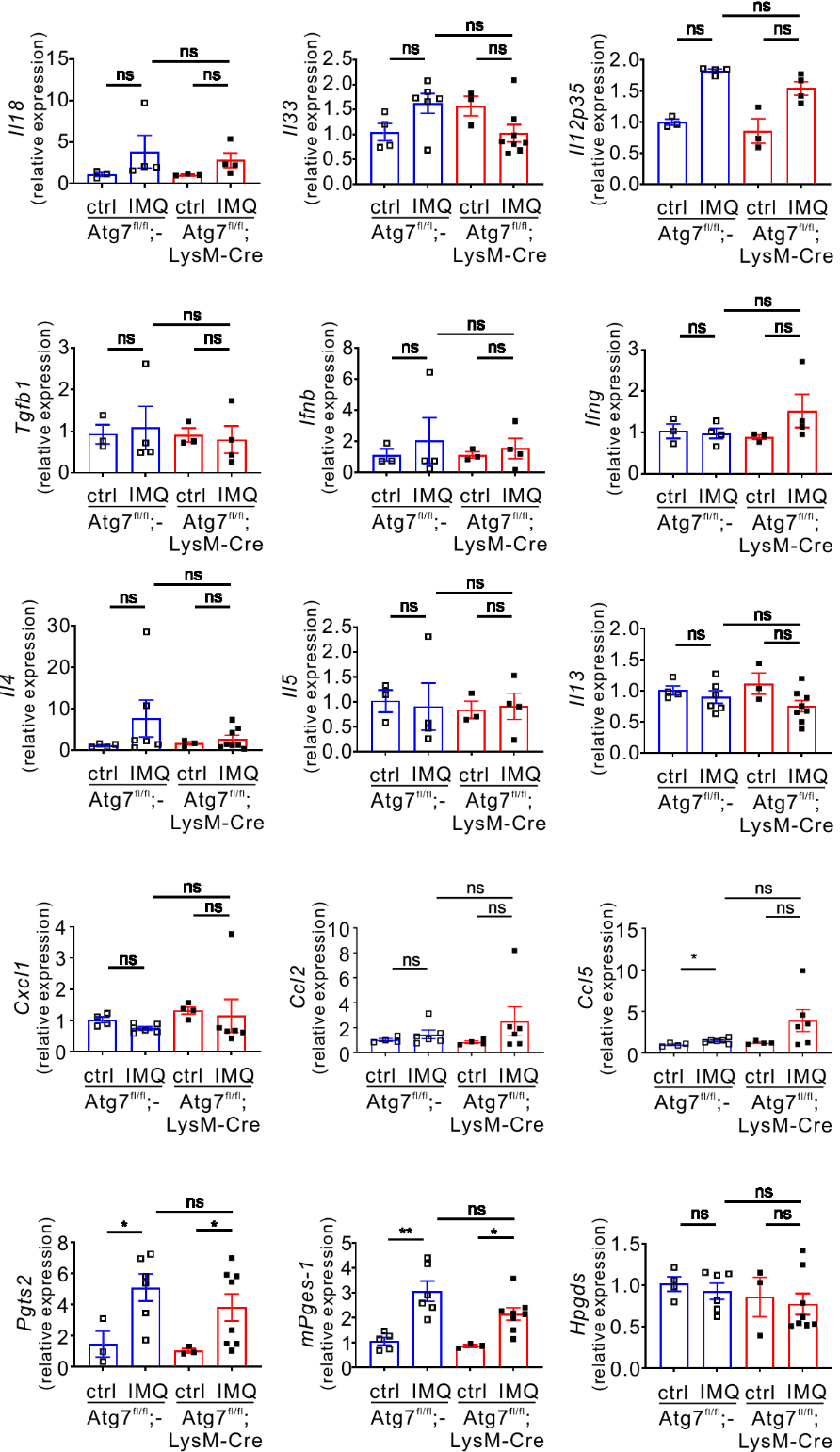


**Supplementary figure 1. Effect of autophagy deficiency on the expression of genes related to inflammation and allergy.**

Effect of autophagy deficiency in myeloid cells on the mRNA expression levels of cytokines related to psoriasis pathogenesis. Relative mRNA levels corresponding to the indicated proteins, as determined by qRT-PCR and normalized to β-actin. All dots indicate the number of samples. All bars indicate mean ± SEM. *P < 0.05 and **P < 0.01, Mann-Whitney U-test.


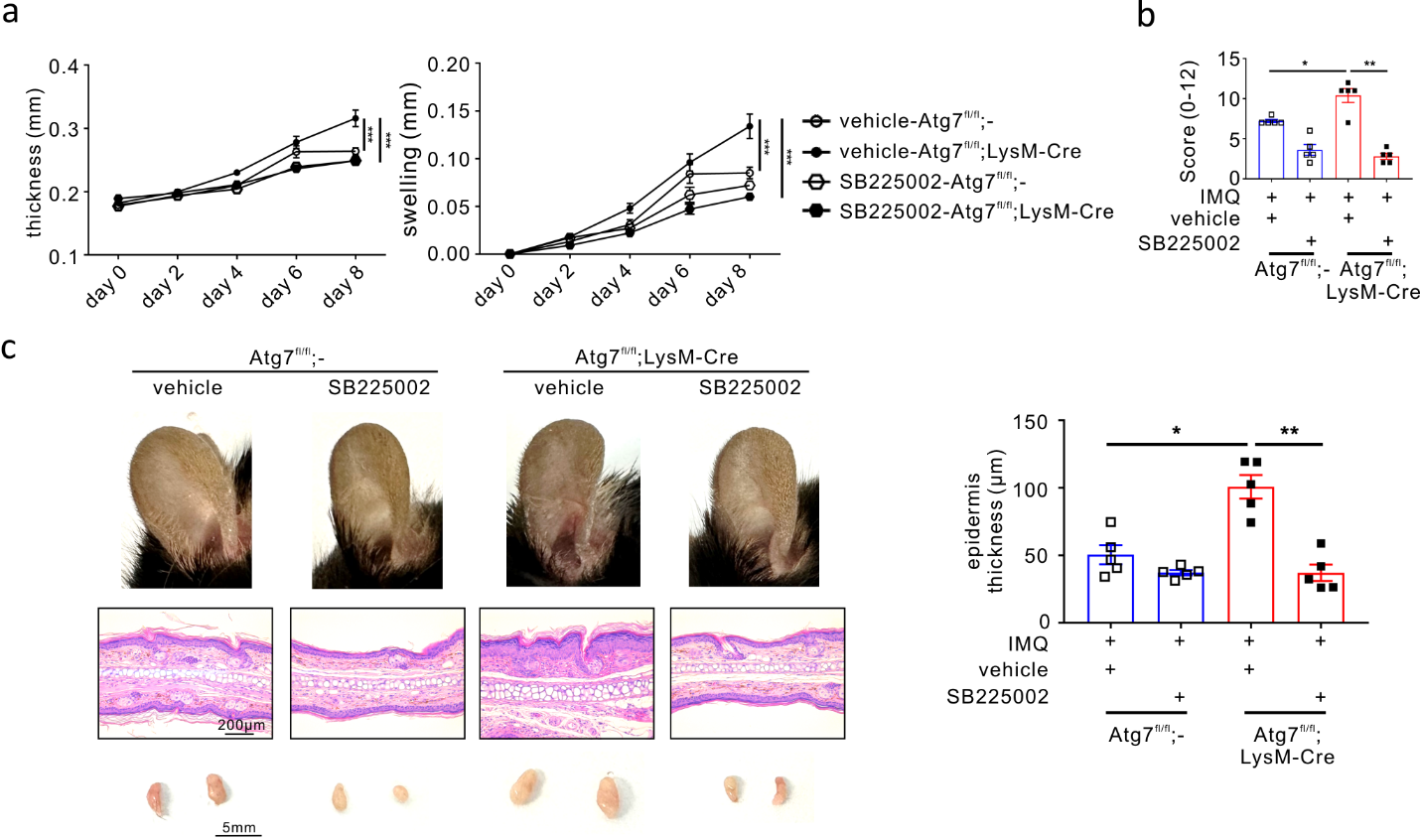


**Supplementary figure 2. Effect of CXCR2 receptor blockade on psoriatic skin inflammation.**

An antagonist of CXCR2 (SB225002) or vehicle was applied onto the ear of *Atg7*^fl/fl^ or *Atg7*^fl/fl^;LysM-Cre mice every day prior to IMQ treatment. Mice were sacrificed at the end of the experiment, and tissues were harvested for each analysis.

(**a**) Ear thickness and swelling were measured every other day (n = 5 per group) ***P < 0.001, two-way ANOVA.

(**b**) Cumulative clinical score (erythema, scaling, and thickness) on a scale from 0 to 12. All bars indicate the mean ± SEM. *P < 0.05 and **P < 0.01, Mann–Whitney U-test.

(**c**) Representative ear images (top), H&E-stained histological images (middle), and draining lymph nodes (bottom) from each mouse group. The bar chart shows the epidermal thickness of ear tissue. All dots indicate the number of samples. All bars indicate the mean ± SEM. *P < 0.05 and **P < 0.01, Mann–Whitney U-test.


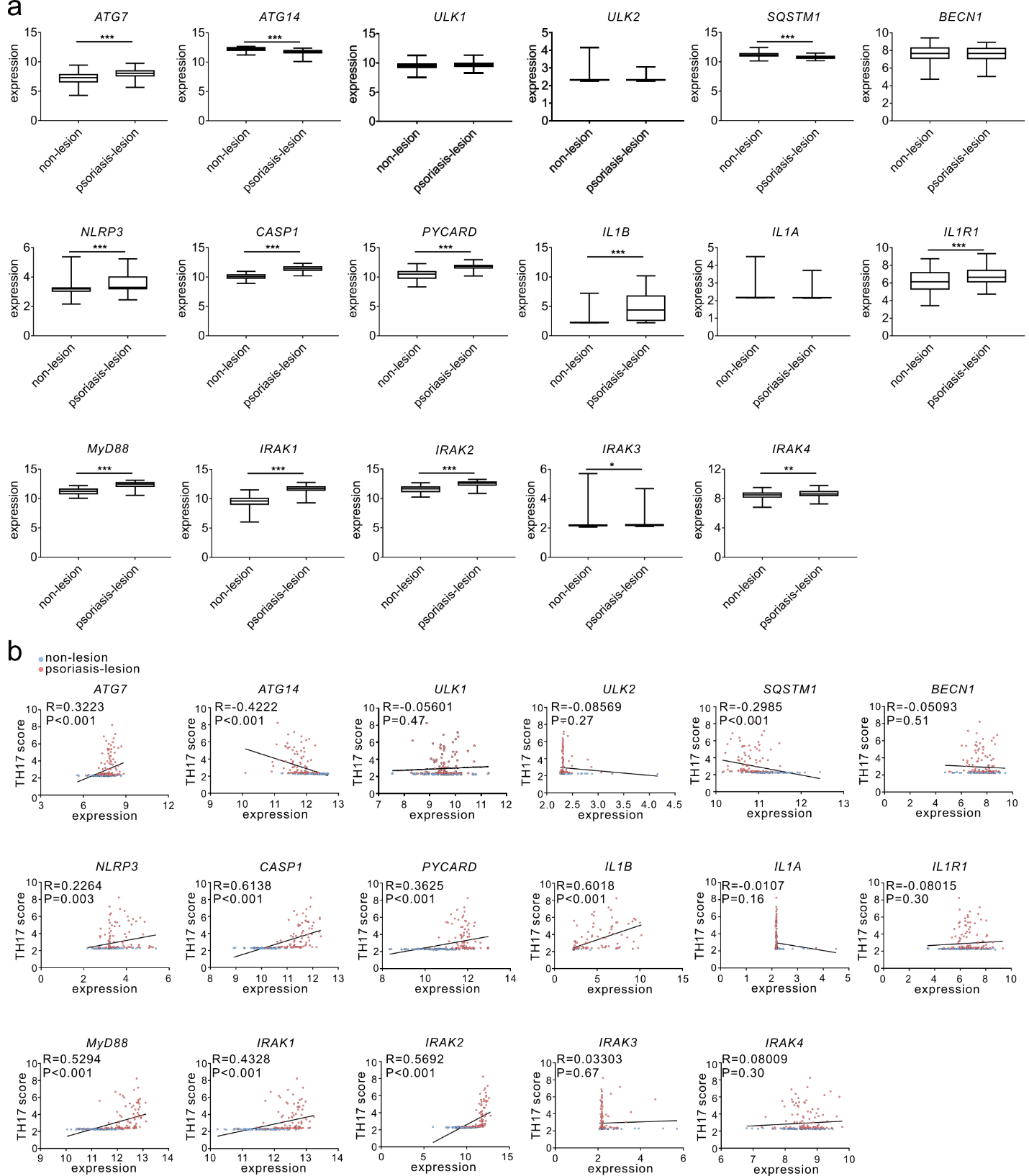


**Supplementary figure 3. Gene expression analysis in human psoriatic skin biopsy specimens using another dataset.**

(**a**) Gene expression profiles were examined in normal healthy non-lesion (NN), patients with psoriatic non-lesion (NP), and patients with psoriatic lesion (PP) using the microarray gene expression dataset (GSE30999).

(**b**) Correlation analysis between target genes and TH17 score. The TH17 score was generated by averaging expression level of IL17A, IL17F, IL22, IL23A, IL23R, and IL12B genes. Blue and red dots indicate patients with psoriatic non-lesion (non-lesion) or patients with psoriatic lesion (psoriasis-lesion), respectively. P-values were calculated by using Mann-Whitney U-tests (a) or the Spearman correlation test (b).
